# Diagnostic accuracy of Pluslife MiniDock MTB on tongue swabs in sputum scarce people with presumptive TB: a retrospective analysis

**DOI:** 10.21203/rs.3.rs-6225528/v1

**Published:** 2026-03-23

**Authors:** Loren Rockman, Shima Abdulgader, Stephanie Minnies, Daphne Naidoo, Arthur Chiwaya, Welile Nwamba, Anna Okunola, Grant Theron

**Affiliations:** Stellenbosch University; Stellenbosch University; Stellenbosch University; Stellenbosch University; Stellenbosch University; Stellenbosch University; Stellenbosch University; Stellenbosch University

**Keywords:** MiniDock MTB, sputum-scarce, tuberculosis, tongue swabs

## Abstract

MiniDock MTB (Pluslife, China) is a near-point-of-care swab-based tuberculosis test. Most data are from people who can expectorate sputum. In 86 sputum-scarce symptomatic adults, retrospective testing detected 53% (95% CI 28–77) of tuberculosis; halving the amount missed if only sputum tests were available.

## Main text

Sputum is a bottleneck to expanding access to tuberculosis (TB) molecular testing (1), a public health priority. Promising swab-based tests, which can be done on tongue swabs or sputum-dipped swabs, are endorsed by the World Health Organization (WHO) (2). However, most data are from people who can expectorate sputum and are hence already testable using existing platforms. The biggest incremental benefit of tongue swabs (TSs) will be in people ordinarily unreachable by sputum tests.

We showed TSs tested with Xpert MTB/RIF Ultra (Ultra; Cepheid, USA) could double the number of sputum-scarce ART-initiators diagnosed compared to if sputum alone was tested (3). Furthermore, a recent study in hospitalised sputum-scarce people in China found a in-house nucleic acid amplification test (NAAT) on TSs could detect 80% of TB that would otherwise require a bronchoscopy to diagnose (4). Lastly, in a multicounty evaluation of MiniDock MTB (Guangzhou Pluslife Biotech, China), the only near-point-of-care WHO-endorsed swab-based NAAT, TS diagnostic yield was non-inferior to sputum (5). We are unaware of MiniDock MTB evaluations exclusively in sputum-scarce people.

This work was approved by the Health Research Ethics Committee of Stellenbosch University (M20/06/017, M21/10/022), City of Cape Town (9517) and Western Cape Department of Health (202203_035). Written informed consent was obtained. We identified flocked swabs (FLOQSwabs code 520C; Copan Italia S.p.A., Brescia, Italy) and paired raw induced sputum stored at −20°C from people ≥ 12 years with presumptive TB recruited in CAGE-TB (Clinicaltrials.gov NCT05317247) or SeroSelectTB (Clinicaltrials.gov NCT04752592) in Cape Town, South Africa from primary care facilities prior to treatment initiation. All people had been asked to expectorate sputum with coaching and had sputum induced if they could not expectorate at least one sputum ≥ 1 ml.

Participants were classified as having TB using a microbiological reference standard (MRS) if, from a single Ultra and/or single MGIT960 culture, induced sputum was Ultra-positive for *Mycobacterium tuberculosis* complex. Participants not having TB had ≥ 1 negative result with no positive induced sputum Ultra or culture result. Participants missing a culture or Ultra result were, if other results negative, classified as not having TB.

TSs from 86 people recruited between 04/07/2022–26/03/2025 were identified, of which 80 (93%) had paired induced sputum. After a median (interquartile range) storage duration of 10 (5–23) months, TSs were tested with MiniDock MTB with a minor protocol deviation to accommodate stored swabs’ shorter breakpoint (30 vs. 80 mm for Pluslife-supplied swabs; **Supplementary text pg. 2**) as collection was prior to MiniDock MTB availability. The rest of the procedure was per the manufacturer’s instructions (6), as was testing of induced sputum with the addition of homogenization using vortexing for 60 s prior to dipping of the swab.

STARD guidelines were followed (**Supplementary text pg. 4**) (7). Diagnostic accuracy metrics were calculated using Excel (Microsoft, Redmond, USA) and compared using prtesti (8) and Fisher’s exact test (9) in STATA version 16.0 (StataCorp, Texas, USA). Diagnostic yield (DYT, diagnostic yield in those tested; DYD, diagnostic yield in those diagnosed) was calculated (10) and are further defined in **Supplementary text pg. 2**.

Twenty-two percent of people (19/86) had TB per the MRS and 20% (17/86) had previous TB. The median (IQR) age was 35 (26–46), 63% (54/86) were female, and 31% (27/86) were people living with HIV (PLHIV).

**Figure 1:**
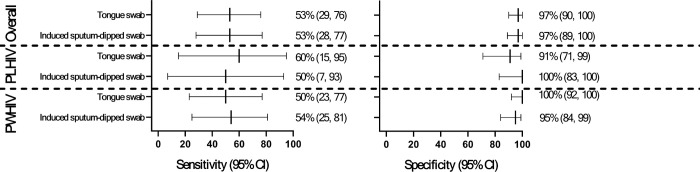
Forest plot of MiniDock MTB sensitivity and specificity on tongue and induced sputum-dipped swabs in sputum scarce people, overall and stratified by HIV. Performance was similar between the two sample types and across different groups, however reduced specificity was observed for tongue swabs in people living with HIV (PLHIV) compared to people without HIV (PWHIV).

TSs and induced sputum-dipped swabs had similar sensitivity [53% (95% confidence interval: 29–76) vs. 53% (28–77); p = 0.985] and specificity [97% (90–100) vs. 97% (89–100); p = 0.950] compared to the MRS (Fig. 1, Table 1). Sensitivity and specificity for each sample type did not differ by previous TB or HIV statuses, however, for TSs, specificity was reduced in PLHIV compared to those without HIV [91% (71–99) vs. 100% (92–100); p = 0.040]. TSs and induced sputum-dipped swabs had similar DYTs (13%, 10/80) vs. (14%, 11/80) and DYDs (43%, 10/23) vs. (48%, 11/23). The proportion TS and induced sputum-dipped swab -positive by induced sputum Ultra semi-quantitation category are in **Supplementary Table 1.**

Our key finding is TS testing using MiniDock MTB in people who - even in a well-resourced research environment - could not expectorate sputum would approximately halve the amount of sputum-scarce TB missed. In other words, TS sensitivity estimates reported here, which are lower than other reports (11) that included primarily sputum productive people with relatively advanced disease, are most appropriately compared to the typical programmatic scenario where sputum-scarce people are generally completely unable to access a rapid molecular test.

Our findings also suggest sensitivity between TSs and induced sputum are similar, which make sputum induction harder to justify in programmatic care, unless perhaps if a more sensitive test (for example, culture) is applied. Notably, MiniDock MTB sensitivity in induced sputum was lower than a prior report (11) that used expectorated sputum, potentially reflecting biomass dilution due to saline. We also identified diminished specificity in PLHIV compared to people without HIV and this appeared independent on previous TB (a cause of false-positivity in sputum NAATs) (12), possibly reflecting oral mycobacterial carriage. Long term follow-up of such “false-positive” people are needed.

Our study had strengths and limitations. To rapidly generate data submittable to a WHO call for data in 2025, we retrospectively tested samples and, for TSs, this necessitate a minor deviation from the instructions-for-use, however, this was done in consultation with the manufacturer. Prospective data from sputum-scarce people, which we are generating, is needed.

In summary, our data suggest MiniDock MTB on TSs is a viable alternative to testing induced sputum, which is seldom available in programmatic conditions, and this can at least halve the amount of sputum-scarce TB missed.

## Supplementary Material

Supplementary Files

This is a list of supplementary files associated with this preprint. Click to download.

• BriefreportSupplementaryresults.docx

## Figures and Tables

**Table 1:** Diagnostic accuracy of MiniDock MTB on tongue swabs and induced sputum-dipped swabs compared to the MRS for the detection of TB stratified by HIV, and separately, previous TB status. Data is %, (95% CI), and n/N.

	Overall^β^ (n = 86)		HIV-negative (n = 59)		HIV-positive (n = 27)	
	Sensitivity	Specificity	Sensitivity	Specificity	Sensitivity	Specificity
**Tongue swab**	53 is(29, 76) 10/19	97 (90, 100) 65/67	50 (23, 77) 7/14	100 (92, 100) 45/45	60 (15, 95) 3/5 p = 0.701*	91 (71,99) 20/22 p = 0.040*
**Induced sputum-dipped swab**	53 (28, 77) 9/17 p = 0.985^€^	97 (89, 100) 61/63 p = 0.950^€^	54 (25,81) 7/13 p = 0.842^€^	95 (84, 99) 41/43 p = 0.143^€^	50 (7, 93) 2/4 p = 0.640^€^ p = 0.720*	100 (83, 100) 20/20 p = 0.167^€^ p = 0.327*
	Overall^β^ (n = 86)		No previous TB (n = 69)		Previous TB (n = 17)	
**Tongue swab**	53 is(29, 76) 10/19	97 (90, 100) 65/67	63 (35, 85) 10/16	96 (87, 100) 51/53	0(0,71) 0/3 p = 0.087*	100 (77, 100) 14/14 p = 0.461*
**Induced sputum-dipped swab**	53 (28, 77) 9/17 p = 0.985^€^	97 (89, 100) 61/63 p = 0.950^€^	60 (32, 84) 9/15 p = 0.886^€^	96 (86, 100) 47/49 p = 0.936^€^	0 (0, 84) 0/2 p ≤ 0.999^€^ p = 0.206*	100 (77, 100) 14/14 p ≤ 0.999^€^ p = 0.442*

Within row p-values: *HIV-negative vs. HIV-positive / No previous TB vs. previous TB

Within column p-values: ^€^tongue swab vs. induced sputum swab

Abbreviations: CI, confidence interval; MRS, microbiological reference standard

β Six people had no MiniDock MTB induced sputum swab result

Two sputum Ultra error results were reported and upon repeat Ultra testing done, resolved to onebeing MTB detected and the other, MTB not detected
